# Sense of coherence of reindeer herders and other Samis in comparison to other Swedish citizens

**DOI:** 10.3402/ijch.v72i0.20633

**Published:** 2013-07-10

**Authors:** Agneta Abrahamsson, Ulrika Lindmark, Arne Gerdner

**Affiliations:** 1 School of Health Sciences, Jönköping University, Jönköping, Sweden; 2 University College of Kristianstad, Kristianstad, Sweden; 3 Mid Sweden University, Östersund, Sweden

**Keywords:** Sami population, non-Sami population, survey, sense of coherence, general resistance resources, health determinants, social support, threats to reindeer herding, Sami life form, meaning of reindeer herding

## Abstract

**Background:**

Samis are indigenous people in north Europe. In the territory called Sápmi (Lapland), reindeer herding is the traditional base for the Sami economy. The relation between living conditions and positive health of the Swedish Samis has been sparsely studied. As health is closely linked to sense of coherence (SOC), an understanding of the background factors to SOC may contribute knowledge that might be useful in promoting living conditions and health.

**Methods:**

The study examines relations between the level of SOC and background factors from surveys in a Sami population (n=613) in comparison to a non-Sami population (n=525) in Sweden, and in comparison between 2 subsamples of Samis, that is, herders and non-herders.

**Results:**

There are more similarities than differences between the Sami and non-Sami populations. However, dividing the Sami population, reindeer herders had significantly lower SOC, and in specific the subcomponent manageability, that is, less ability to use available resources to meet different demands in life, compared to non-herders.

**Conclusions:**

In addition to age and health, predictors of SOC are related to the life form of reindeer husbandry and the belonging to the herding community.

The Samis are indigenous people in northern Fenno-Scandinavia, a territory called Sápmi (Lapland). The estimated number of Samis is unclear due to disputed definitions and a lack of ethnic-specified censuses for many decades (1). One estimate based on criteria of descendants of Samis in 1945 claims that the total number of Samis would be between 50,000 and 65,000 (2,3).

Reindeer herding is the traditional base for the Sami economy. It is important not only for the herders themselves but also for the economy in the whole Sápmi territory because of its attraction to tourists coming to the area. Traditional reindeer herding implies a nomadic way of life when following large herds of reindeers from the fell areas (mountains above the tree line) in the summer to the forests in the winter (2). Modern herding implies driving snow-scooters and motorcycles in rough terrain and harsh climate. The living conditions for reindeer herders have been worsened due to the majority population’s, that is, non-Sami Swedes, exploitation of land and nature resources, resulting in conflicts of land use especially during winter when the pasture of reindeers are in the forest land (2–5). Furthermore, the use of terrain motorcycles, snow mobiles and helicopters is expensive (2). The reindeers are also sensitive to increased pressure from more numerous predatory animals (3). Legal conflicts concerning these issues may have contributed to the feelings of being subjected to outer pressure, that is, pressure that arises form conflicts of interests between the Sami and the non-Sami Swedish society (6). Of the estimated 17,000–20,000 Sami individuals living in Sweden, 8,400 are registered as Samis as voters for the Sami Parliament, and 2,500 are still more or less dependent on reindeer herding as income for living (6,7).

The relation between living conditions and health of the Swedish Samis has been sparsely studied (8,9). A recent review on the health of the Samis describes the physical risks and problems for reindeer herders, mostly men (10). According to this report, both men and women rate their social, physical and mental quality of life as lower compared to other Swedish citizens. The sample of that study was limited to the herding population, not reflecting the situation for the majority of the Samis. Mental health problems amongst reindeer herders were in a thesis (8) seen as linked to the specific life form and life style of herding which is challenged from internal pressures within Sami society that arise from scarce economy and internal conflicts between reindeer herders and other Samis, and outer pressures from non-Sami Swedish society and economy. The specific health problems found especially among the reindeer-herding Samis, appear to originate from isolation of the reindeer husbandry within the Swedish economy, but also from poor knowledge about the reindeer-herding Sami culture within the majority population of non-Sami Swedes (11). As non-herding Samis were not included in previous studies, the extent to which problems are specific to the herding community is not clear.

Sense of coherence (SOC) is an often-used concept to understand health-promoting living conditions (12). The theory behind the SOC builds on beliefs that promotion of health can be understood from other factors than just prevention and curing diseases (13,14). An individuals’ level of SOC is an expression of a persons’ ability to use available internal and external resources in a healthy direction. The concept consists of 3 components: (a) *comprehensibility* is the extent to which the individual perceives the demands in life to be clear, ordered and structured; (b) *manageability* is how easily the individual could find resources for coping; and (c) *meaningfulness* is the way the individual regards life to be purposeful and demands worthwhile of the investment of emotions (12).

The level of SOC, according to Antonovsky (12), originates from the individuals’ life in its whole context. Although different life experiences available to the individual may contribute to the development of SOC through the whole life span, it should be noted that the experiences in the first decades are most important (15). These experiences can be positively internalized as Generalized Resistance Resources (GRR), which may be defined as “any characteristic of the person, the group, or the environment that can facilitate effective tension management” (16, p. 99). Examples of GRR could be cognitive, emotional, attitudinal, interpersonal–relational and socio-cultural resources as well as material security (17). The levels of SOC and GRRs are interdependent. If an individual’s early experiences were canalized into the development of GRRs, (s)he will develop a strong SOC. At the same time, a strong SOC may facilitate the use of GRRs at her/his disposal as well as strengthening the further development of GRR. GRRs are not only linked to individual resources, for example self-efficacy and intelligence, but also to external resources from social groups, networks or a feeling of a safe and secure environment (16).

A Finnish cross-sectional study concluded that the level of SOC is positively related to psycho-social wellbeing due to GRRs, such as close relationships, social support, and working conditions and employment status in later life (18). Although circumstances early in life are important in influencing the development of the SOC level, other background factors such as health, wealth and material pre-conditions, cultural stability, social support and ego strength improve SOC later in life (18). In a review of research in different populations, the level of SOC was found to be strongly related to perceived health, especially for those with initial high SOC (17). Health as a GRR may influence SOC, but SOC is often also studied as a factor influencing health. Although the level of SOC does not alone explain all aspects of health, it contributes to the development and maintenance of people’s health (17). Longitudinal studies in different countries have found that the individuals’ levels of SOC can predict health (17,19,20). SOC is inversely related to dramatic events such as personal life crises and stress and is positively related to social support (17,21).

The relations between the level of SOC and GRRs later in life, for example occupation, social support, socio-economic position and perceived strain at work (18,21) indicate that investigating predictor background factors may contribute knowledge useful in health promotion. The aim in this study is to examine the level of SOC and resources with possible impact on health, that is, education, cohabiting, work status, health, belonging and attitudes to reindeer herding, as predictors to SOC in a Sami population in Sweden in comparison to a population of non-Sami Swedes, and in comparisons between two subsamples of Samis, that is, herders and non-herders.

## Method

The study consists of 2 samples.

### Sami sample

The Sami sample is based on a representative survey of Samis registered as voters to the Sami Parliament and living in 3 Swedish counties, that is, Norrbotten, Jämtland and Stockholm counties. It is part of a larger research project on the identity and psychosocial health of Swedish Samis. About 400 Samis of each county were selected through randomization. Of 1,192 mailed questionnaires, 1,148 persons in the randomized sample received questionnaires, and 613 replied with completed forms (53.4%). Questionnaires were in Swedish, which is now the common language for most Swedish Samis. Respondents who preferred one of the Sami languages were offered professional translations. One person made this claim. The questionnaires were comprehensive, and included questions on Sami life, welfare and health, among these the Swedish version of Antonovsky’s 13-item SOC scale.

### Non-Sami sample

We also evaluated a stratified random sample of 910 non-Sami individuals from Jönköping, Sweden, a medium-sized city with around 125,000 inhabitants (in 2003) in the south of Sweden where few Samis live. The sample was selected in 2003 from the county administrative board for a study on dental health (22). The sample consisted of 130 age-stratified randomly selected subjects in each of the age groups 20, 30, 40, 50, 60, 70 and 80 years – a total of 910 individuals. Of these, 10% had a non-Swedish ethnical background, which was similar to the Swedish population in 2003 (23). The individuals were asked to fill in a self-report questionnaire in which questions were asked about their attitudes towards and knowledge of teeth and dental care habits. It also contained the same Swedish version of the SOC scale. Of the individuals who showed up at an oral examination (n=589), finally 525 individuals answered the SOC questionnaire and were thus included in the analyses (57.7%).

### Ethics

The Sami Study was approved by the Regional Ethical Review Board in Umeå (ref. no: 08-158Ö) and got support from the Sami Parliament (ref. no: K 2007-250), while the dental study was approved by the Regional Ethical Review Board in Linköping (ref. no: 02-376). The combined data set for this study was constructed based on non-identifiable subsets using only the variables needed for this specific study.

### Instruments

The SOC 13-item questionnaire consists of 3 interrelated components, comprehensibility, manageability and meaningfulness (12). Five items measured comprehensibility (e.g. In an unfamiliar situation, do you feel you just do not know what to do?), 4 items measured manageability (e.g. How often do you feel you are not able to cope?) and 4 items measured meaningfulness (e.g. How often do you feel daily life is meaningless?). Every item was scored on Likert scales, ranged from 1 to 7 points. The scales were constructed as the sum of the items.

The total range of the SOC scale is 13–91 points (12). High scores indicate a strong SOC. The mean value of the SOC-13 has varied from 35 to 77 in different groups (19). The questionnaire has been shown to produce acceptable results in terms of both high validity and reliability. From an extensive review, Eriksson and Lindström (24) concluded that the SOC scale seems to be a reliable, valid and cross-culturally applicable instrument. The brief SOC-13 was tried in the present population, subscale by subscale as well total scale, using principal component analysis, giving showing acceptable explained variances (R^2^>0.51 for all subscales and 0.40 for the combined total scale). All loadings were above 0.45. Separate analyses on the 2 subsamples gave similar results. Internal consistency in the present population for the SOC and all its subscales were tried with Cronbach’s alpha and ranged from 0.70 to 0.88 in the Sami sample and from 0.65 to 0.86 in the non-Sami sample. Thus, all SOC scales showed to be homogeneous and with acceptable to satisfactory consistency in the samples studied (25).

Two scales constructed for the Sami study and used only in the Sami sample concerned attitudes towards reindeer herding. They were based on 3 items, each with Likert scales (0–10). The index scales were constructed as means of the items of each scale.

- *Importance of reindeer herding* is based on questions on the responsibility of the reindeer herders to uphold the tradition, the prospects of reindeer herding as a sustainable industry and the importance of reindeer herding for the culture of the Sami people. The index scale varied from 0 to 10, with a mean of 7.2 (SD=2.0) and median 7.7. The explained variance based on these items was high (R^2^=0.63, as were the factor loadings of each item (0.66–0.89) and the internal consistency was acceptable (α=0.68) (25).
- *Threats to reindeer herding* included questions on threats to reindeer herding because of changes in the landscape, threats due to the policy to preserve predators (wolves, wolverines, lynxes and bears) and threats concerning the legal position of reindeer herding versus foresters (for the winter grazing lands). The index scale varied from 0 to 10, with a mean of 7.8 (SD=2.2) and median 8.3. The explained variance based on these items was high (R^2^=0.73, as were the factor loadings of each item (0.64–0.80) and internal consistency was satisfactory (α=0.82).

### Relational statistics

Data analysis was carried out using the statistical software Statistical Package (SPSS) version 19. Descriptive statistics were used to obtain means (SD). Significance of differences was tested by using student *t*-test for independent groups in normally distributed variables; the non-parametric Mann–Whitney U-test for skewed distributions, and Chi-square in categorical variables.

Multivariate linear regression was used in order to predict SOC from demographics, work and education status and perceived health. Models were created using backward deletion. The items used as predictors were education, cohabitating, work status, health and belonging to the Sami or non-Sami Swedish population. In the Sami population, 2 additional factors were tried: importance of reindeer herding and threats to reindeer herding.

## Findings

There are some differences between the 2 samples. Demographic differences concerning age and gender approach do not reach statistical significance (since 0.05<p<0.1). Samis are older, and Sami women responded more than Sami men, while responses were equal between genders among non-Samis.

There are some differences in possible background factors that could be related to the total SOC level. The Samis have regular work significantly more often than non-Samis but are also more often pensioners’ due to health-related issues. Non-Sami people are more likely to be students or have some other occupation that prevents them from working in wage- and salary-based labour. Samis more often than non-Samis declare themselves to have health problems. Additionally, Samis are somewhat more often married or cohabitating than non-Samis, although this did not reach statistical significance (0.05<p<0.1). Thus, there are several background factors that differ and need to be accounted for in the analysis of how they might have a bearing on the SOC and how it varies between the two samples.

There is however no significant difference between Samis and non-Samis on the total SOC score, but a difference in score is found in one of the 3 subcomponents. Samis score higher than non-Samis on comprehensibility – the extent to which the individual perceives the demands in life to be clear, ordered and structured (Table I).

**Table I:** Comparisons between Samis and non-Samis on sense-of-coherence with its 3 subscales

	n	Samis	Non-Samis	p
Sense-of-coherence (total)	601/525	71.0 (12.1)	70.0 (11.5)	0.177
Comprehensibility	601/525	26.5 (5.4)	25.8 (5.3)	0.027
Manageability	601/525	21.5 (4.3)	21.3 (3.9)	0.566
Meaningfulness	601/525	23.0 (4.1)	22.9 (3.9)	0.662

SOC=sense-of-coherence.

However, these findings must be studied in relation to differences in background factors to the total SOC level between populations as described in Table II. This is done in multivariate linear regression models, with total SOC and its subcomponents as dependent variables and with background factors in Table II, together with being Sami or not, as independent variables that are possible predictors. Work status is then collapsed into the dichotomous variable *Have regular work* (yes/no). The optimal multivariate models are shown in Table III, with the order of independent variables given according to the standardized regression coefficient Beta, which indicates the relative strength of the predictor.

**Table II:** Description of the study populations, Samis and non-Samis

	n	Samis	Non-Samis	p
Age, mean (SD)	609/525	49.9 (15.2)	48.0 (19.2)	0.068 (a)
Gender, female (%)	613/525	55.3	49.7	0.060 (b)
Country of birth, Sweden (%)	613/514	96.4	89.1	0.000 (b)
Married/co-habitating (%)	613/512	69.0	62.1	0.015 (b)
Education (%)	613/514			0.002 (b)
Less than 9 years compulsory education		9.1	16.0	0.574 (c)
Nine years compulsory education		10.2	8.2	
At least 1 year additional vocational education		22.2	2.1	
Secondary high school (at least 12 years of education)		24.1	49.6	
Academic exam		34.4	24.1	
Work status (%)	602/514			0.000 (b)
Have regular work		69.4	57.2	
House wife/house man		0.5	0.8	
Pensioner due to age		19.6	22.4	
Pensioner due to sickness/disabled		5.1	2.7	
Out of work		2.7	3.5	
Studying		2.2	8.6	
Else		0.5	4.9	
Declares oneself as “healthy” (%)	605/514	67.3	75.9	0.002 (b)

Significance tested with (a) Student t-test for independent groups; (b) Chi-square; (c) Mann–Whitney U-test.

**Table III:** Multivariate regressions on sense-of-coherence and its 3 subcomponents

Model	P for models	R^2^	Independent variables	B	SE of B	Beta	P for independent variables
Sense-of-coherence (total)	0.000	0.16	Age	0.25	0.02	0.36	0.000
			Healthy	5.83	0.75	0.22	0.000
			Education	0.98	0.22	0.14	0.000
			Cohabitating	3.01	0.71	0.12	0.000
			Have regular work	1.69	0.77	0.07	0.029
Comprehensibility	0.000	0.13	Age	0.11	0.01	0.35	0.000
			Healthy	2.19	0.34	0.19	0.000
			Cohabitating	1.13	0.33	0.10	0.001
			Education	0.31	0.10	0.10	0.002
			Have regular work	0.70	0.36	0.06	0.048
Manageability	0.000	0.14	Age	0.08	0.01	0.34	0.000
			Healthy	1.94	0.26	0.21	0.000
			Cohabitating	1.09	0.25	0.13	0.000
			Education	0.23	0.08	0.09	0.002
			Have regular work	0.49	0.27	0.06	0.075
Meaningfulness	0.000	0.11	Age	0.06	0.01	0.26	0.000
			Healthy	1.67	0.26	0.19	0.000
			Education	0.44	0.08	0.18	0.000
			Cohabitating	0.80	0.25	0.09	0.001
			Have regular work	0.50	0.27	0.06	0.064

SOC=sense-of-coherence.

Optimal models after backward deletion with Sami ethnicity, age, gender, country of birth, cohabitating status, education, work and regarding oneself as “healthy” as initial predictor candidates (background factors).

All models are fairly similar and highly significant (p<0.001), although they explain a modest share of the variance of the respective independent variable (R^2^=0.11–0.16). All models show age and perceived health as the strongest predictors, and work status as the weakest (and sometimes not reaching significance), while cohabitating and education are in between these 2 (and shifting places). All models excluded gender, being Sami and country of birth as non-significant variables. Thus, being Sami seems not to have any direct bearing on total SOC or its subcomponents.

The Samis are not a homogeneous population. One of the most obvious differences is whether or not the Samis continue their traditional life related to reindeer-herding. The question arises, if this life form is a significant predictor of the individuals’ SOC? Table IV presents 2 groups of Samis, those who are, or have been, directly involved in the reindeer industry as herders, and those who never were.

**Table IV:** Description of 2 Sami populations, reindeer herders (now or previously) and non-herders

	n	Herders (ever)	Non-herders	p
Age, mean (SD)	116/493	52.1 (16.8)	49.3 (14.8)	0.112 (a)
Gender, female (%)	118/495	38.1	59.4	0.000 (b)
Country of birth, Sweden (%)	118/495	94.9	96.8	0.331 (b)
Married/co-habitating (%)	118/495	70.3	68.7	0.727 (b)
Education (%)	117/490			0.000 (b, c)
Less than 9 years compulsory education		20.5	6.3	
Nine years compulsory education		22.2	7.3	
At least 1 year additional vocational education		12.8	24.5	
Secondary high school (at least 12 years of education)		25.6	23.7	
Academic exam		18.8	38.2	
Currently in regular work (%)	118/495	65.3	68.9	0.446 (b)
Declare oneself as “healthy” (%)	117/488	62.4	68.4	0.210 (b)

Significance tested with (a) Student’s t-test for independent groups; (b) Chi-square; (c) Mann–Whitney U-test.

Two important differences between herders and non-herders within the Sami population appear. Herders as opposed to non-herders are mostly male and have lower education. No other differences are significant between herders and non-herders in the Sami population. For Samis, some other relevant scales that might be seen as background variables to the SOC are available. These are indices on how the person rates the importance of and the threats to reindeer herding. These 2 scales as well as total SOC and its 3 subcomponents are compared between herders and non-herders in Table V.

**Table V:** Comparisons between herders and non-herders on sense-of-coherence with its 3 subcomponents, importance of and threats to reindeer herding

	n	Herders	Non-herders	p
Sense-of-coherence (total)	114/487	68.4 (13.8)	71.6 (11.6)	0.022
Comprehensibility	114/487	25.7 (6.0)	26.7 (5.2)	0.066
Manageability	114/487	20.2 (4.9)	21.8 (4.0)	0.002
Meaningfulness	114/488	22.5 (4.4)	23.1 (4.0)	0.152
Importance of reindeer herding	115/485	8.0 (1.5)	7.1 (2.0)	0.000
Threats to reindeer herding	115/484	8.7 (2.0)	7.5 (2.2)	0.000

SOC=sense-of-coherence.

Significance by t-test for independent groups.

Herders score significantly lower than non-herders on total SOC and its subcomponent manageability, and lower on comprehensibility, although the latter does not reach significance. They score higher than non-herders on the possible background variables to the SOC, that is, importance of and threats to reindeer herding.

Each of the 2 groups can be compared to the non-Samis (for ratings, see Table I), who place themselves between the 2 Sami groups on total SOC and all 3 subcomponents. Herders score significantly *lower* on manageability (p=0.021) compared to non-Samis, while non-herders score significantly *higher* on total SOC (p=0.038) and on comprehensibility (p=0.010), and a tendency to score higher on meaningfulness although this does not reach significance (p=0.063).

As the last step, the multivariate regressions to predict total SOC and its subcomponents are repeated using only the Sami population. Possible background variables that could predict an individual’s SOC are the 6 factors found in Table IV, that is, age, gender, cohabitating status, education, regular work, declare oneself as healthy plus the 2 new scales importance of reindeer herding and threats to reindeer herding, as well as the dichotomous variable reindeer herder (or not). These are presented in Table VI.

**Table VI:** Multivariate regressions (backward deletion) on sense-of-coherence and its 3 subscales applied to the Sami population

Model	P for models	R^2^	Independent variables	B	SE of B	Beta	P for independent variables
Sense-of-coherence (total)	0.000	0.17	Age	0.25	0.04	0.31	0.000
			Healthy	5.85	1.00	0.23	0.000
			Cohabitating	3.78	1.01	0.14	0.000
			Importance of reindeer herding	0.73	0.27	0.11	0.007
			Reindeer herder	−3.38	1.21	−0.11	0.006
			Threats to reindeer herding	−0.50	0.23	0.09	0.030
			Have regular work	2.29	1.17	0.09	0.050
Comprehensibility	0.000	0.13	Age	0.11	0.01	0.29	0.000
			Healthy	2.27	0.45	0.20	0.000
			Cohabitating	1.46	0.46	0.13	0.001
			Reindeer herder	−1.07	0.54	−0.08	0.046
Manageability	0.000	0.16	Age	0.09	0.01	0.33	0.000
			Healthy	1.76	0.35	0.19	0.000
			Reindeer herder	−1.43	0.43	−0.13	0.001
			Cohabitating	1.18	0.36	0.13	0.001
			Threats to reindeer herding	−0.19	0.08	−0.10	0.013
			Have regular work	0.91	0.41	0.10	0.026
Meaningfulness	0.000	0.12	Healthy	1.72	0.35	0.20	0.000
			Age	0.05	0.01	0.19	0.000
			Education	0.41	0.10	0.17	0.000
			Importance of reindeer herding	0.31	0.09	0.15	0.000
			Cohabitating	1.20	0.35	0.14	0.001

SOC=sense-of-coherence.

All models are highly significant, although they explain a moderate share of the variances (R^2^=0.12–0.17). As in previous models, we find that age and health are the strongest predictors of the total SOC level and its subcomponents. Cohabitating remains as a predictor in all models. Having regular work remains only in the first model (total SOC), but not for the subcomponents, and education appears now as a predictor only of meaningfulness, but not for the other components. Reindeer herding appears as a negative predictor of SOC, comprehensibility and manageability, but not of meaningfulness. Having a positive view on the importance of reindeer herding is instead a positive predictor of total SOC and of meaningfulness, while seeing strong threats against this industry is a negative predictor of the total SOC level and of meaningfulness.

## Discussion

The study examines the level of SOC and various GRRs as predictors to SOC in a Sami population in comparison to a non-Sami population in Sweden. The main result shows that there are generally more similarities than differences between the Sami and non-Sami populations. The similarities between the 2 populations are confirmed in the multi-regression models, where being a Sami does not have any bearing on the SOC and its subcomponents. Acculturation theories often point out membership of minority ethnic groups to be problematic factors for health and health promotion (26). The Sami reported more health problems than the non-Sami did, and more Sami were pensioners due to health-related issues. Nonetheless, we found no support in this study that Sami ethnicity account for any differences in SOC or its subscales as health-promoting factors. The explained variances of SOC and its subscales are limited, as could be expected in a study of 2 ethnic groups of this kind. More within-group factors, not assessed here, should have an impact on SOC. However, the models are highly significant and consistent. Age and health are the strongest predictors to the level of SOC in all models, independent of population. These findings are in accordance with previous research (17,27). Other predictors in all models are cohabitating, education and, although weaker in significance, having work. These findings are similar to the findings in a Finnish study (18), which concludes that psycho-emotional resources together with socio-economic resources do have a bearing on the level of SOC.

However, it is important to realize that even if a population shows a high total SOC score, there may be differences within a population, for example between age groups and genders. Another recent study shows the limitation of just measuring total SOC in a population in that the distribution of the level of the SOC subcomponents can be complex (27). These results imply that the subcomponents strengths are influenced by different kinds of experiences in life (12). An analysis of the SOC as well as the 3 subcomponents within a population can contribute useful knowledge on the implications to the SOC from different background factors and life experiences.

Therefore, this study also examines 2 important subgroups within the Sami population, herders and non-herders. Differences are shown in total SOC scores as well as in the subcomponents. The subcomponent manageability explains the differences in the total SOC score between herders and non-herders, while comprehensibility is the subcomponent explaining differences between Samis and non-Samis. These results can be interpreted as the subcomponents having different relevance depending on different contexts of the populations. Moreover, non-herders seem to be more similar to the non-Sami population with regards to the SOC. This study demonstrates the importance of considering subgroups within populations, which has also been shown in previous research (27). Not considering this, the conclusions of the results in a population, for example health in relation to SOC, can be misinterpreted and incorrectly generalized.

The reindeer herders within the Sami population score lower total SOC, which is mainly explained by the subcomponent manageability, and less by comprehensibility. Being a reindeer herder is a strong *negative* predictor to the SOC and to the subcomponents manageability and comprehensibility. Furthermore, feelings of threats to the reindeer herding is also a negative predictor to the SOC and to manageability. These results should be seen in the light of the access to land that has been cut successively during the last 100 years because of forestry interests which implies more difficult husbandry of reindeers (4,5). These threats to reindeer herding push demands on extra monitoring and more of working hours, costly feeding of the reindeers and costly equipment (6). Low level of total SOC and the subcomponent manageability may therefore be seen as an implication of environmental changes, worsened life and working conditions for reindeer herders to earn their living. These circumstances may contribute to explaining the previously described health problems among reindeer herders (10).

But why do the reindeer herders continue with this threatened lifestyle? Qualitative studies on Sami life forms found that social support is important in order to resist outer pressure directed against reindeer herding (6,28). Another indicator is the significant relation between cohabitating and the level of SOC. Thus, one important factor may be the acknowledgement that herders get from their nearest social network. Nevertheless, there seems to be an even stronger factor, as indicated by the regression model on meaningfulness. This might seem odd, since there are no significant differences in meaningfulness between herders and non-herders, or between Samis and non-Samis. Still, meaningfulness is the only subcomponent of SOC, to which being a herder is not a negative predictor. Instead, we find that the scoring is high on the importance of reindeer herding which is a significant positive predictor of meaningfulness. Herders rate the importance of reindeer herding higher than non-herders, although many others around the herders also rate this importance as high. Thus, although herders do not in general have more or less meaningful lives compared to others, it seems that herders themselves and those around them, who acknowledge the importance of reindeer herding, get a stronger sense of meaning from this. This interpretation may illustrate a possible reason for staying as a reindeer herder; you continue to be a herder because it gives your life meaning despite the challenges met.

In line with Åhrén (28), our findings illustrate the specific situation of belonging to a reindeer herding community, an experience in which most aspects of life are patterned from the position within the shared community. Thus, being a herder is a way of life rather than an occupation (29). A necessary pre-condition for this way of living is the view of reindeer herding as important and having social support in that. The lifestyle of being a reindeer herder becomes meaningful, despite the low level of manageability due to outside pressure on the industry, and despite lower comprehensibility in relation to the outer world.

The reindeer herders are mostly men with relatively low education. In this model, however, education did not predict higher comprehensibility, but higher meaningfulness. Meaningfulness is also predicted by the importance of reindeer herding, on which herders score high. As these men are often raised in families of origin based on the same way of life, that is, being a reindeer herder (6), they see the importance of reindeer herding as more crucial than formal education (29). The problems of their lower educational level influence their way of life as it limits their ability to comprehend what happens in outer society reflected here as lower comprehensibility.

### Study limitations

One obvious limitation is that data come from surveys, not from prospective data. This is the situation in most studies on SOC, and thus the causal relations, for example between health and SOC, cannot be clearly defined. As with most epidemiological studies, the response rates are limited. Both samples, however, have similar or even better response rates compared to Swedish General Health studies, that is, >50%. Cross-cultural comparison needs an understanding of the meaning of various occupations for the groups at study. To the reindeer herders the occupation was in fact not just a job, but also a total way of life that extends beyond economic stability and one that is a vital part of their identity. The implication of occupation to non-herders and to members of the non-Sami population is probably more complex than shown here, and may need to be studied further.

## Conclusions

The Samis in general are alike the non-Sami population in the level of SOC and its subcomponents. Dividing the Sami population, reindeer herders had significant lower SOC, specifically in the subcomponent manageability, compared to non-herders. Beside age and health as the strongest predictors to SOC, being related to the life form of reindeer husbandry is strongly related to the level of SOC and its subscales. Herding is associated with lower SOC, manageability and comprehensibility, but remains centrally important to a herder’s sense of meaningfulness.
